# Ovarian cancer disease burden decreased in the United States from 1975 to 2018: A joinpoint and age-period-cohort analysis

**DOI:** 10.1097/MD.0000000000036029

**Published:** 2023-12-01

**Authors:** Jiahui He, Qinyong Hu

**Affiliations:** a Department of Oncology, Renmin Hospital of Wuhan University, Wuhan, China.

**Keywords:** age-period-cohort analysis, incidence, incidence-based mortality, joinpoint regression analysis, ovarian cancer

## Abstract

Ovarian cancer (OC) is the leading cause of gynecological cancer-related deaths in the United States. The purpose of this study was to evaluate long-term trends in OC incidence and incidence-based mortality rates (IBM) in the U.S. from 1975 to 2018 and to assess the effects of age, period, and cohort factors on OC incidence and mortality using an age-period-cohort model. We obtained data from the U.S. OC incidence/mortality data from the Surveillance, Epidemiology, and End Results database from 1975 to 2018. Joinpoint regression analysis was used to determine long-term trends and transitions, and an age-period-cohort model was used to quantify the effects of age, period, and cohort parameters on incidence and mortality. In addition, 1990 to 2019 U.S. OC data obtained from the Global Burden of Disease study served as a potential validation set. Between 1975 and 2018, 80,622 new cases of OC and 60,218 deaths from OC were reported in the U.S. The average annual percent change for OC incidence was −1.33 (95% CI: −1.64 to −1.02, *P* < .001), with a significant decrease in incidence at a rate of 7.80% (95% CI: −11.52 to −3.92) per year from to 2015–2018. IBM reached its peak for the U.S. population in 1994, with an age-standardized mortality rate of 6.38 (per 100,000 people). IBM rose first, peaked in 1986, and then declined at a rate of 0.39% (95% CI: −0.66 to −0.12) and 2.48% (95% CI: −3.09 to −1.85) per year from to 1986–2007 and 2007–2018, respectively. In addition, age-period-cohort model analysis showed the highest risk of OC incidence in 1980 to 1984 and the highest risk of OC death in 1985–1989. This study reported a significant decline in OC morbidity and mortality in the U.S. since 1986. In addition, this study analyzed the changes in trends in OC incidence and mortality by race/ethnicity in the U.S. Monitoring trends in OC incidence and mortality by race/ethnicity can help in the development of targeted prevention and treatment measures.

## 1. Introduction

Ovarian cancer (OC) is the fifth leading cause of cancer-related death in women in the United States and the most common gynecological malignancy in.^[1]^ There are 5 different histological types of OC, of which high-grade plasmacytomas are the most common.^[2]^ The risk of OC increases with age, with a sharp rise in incidence after the age of 50 years and an average age of diagnosis of 50 to 70 years.^[3]^ Although OC accounts for only 3% of all cancers in women in the United States, the lack of specific symptoms or effective screening and early detection strategies results in more than 70% of patients with stage III or IV tumors at initial diagnosis and a poor prognosis, even with immediate and aggressive treatment.^[4]^ The 5-year survival rates for patients with stage III and IV OC are 36% and 17%, respectively, and the 10-year survival rates are 23% and 8%, respectively.^[5]^

Sopik et al^[6]^ reported that age-adjusted incidence and mortality rates of OC in the U.S. decreased by 26% and 23%, respectively, between 1975 and 2011. The incidence of epithelial OC in the U.S. declined or remained stable across races/ethnicities from 2000 to 2013.^[7]^ Liao et al^[8]^ divided the OC data from the Surveillance, Epidemiology, and End Results (SEER) database into 3 time periods, 2001–2005, 2006–2010, and 2011–2014, and the age-adjusted incidence rate of OC decreased from 5.31 to 5.08 and then to 4.86. Based on data from the SEER database, Phung et al^[9]^ observed a decrease in the incidence of high-grade plasmacytic OC between 1992 and 2019. Because the primary population in most OC studies is white women, there is inconsistency in published studies regarding racial differences in OC survival.^[10]^ In the U.S, black women are generally less likely to develop OC than white women, but have a higher risk of OC-related death.^[11–14]^ A large retrospective study of 172,849 patients diagnosed with OC cancer from 2001 to 2009 (including nearly 14,000 black patients) by Stewart et al^[15]^ provided strong evidence for racial disparities, with black women consistently showing lower survival rates than white women. The data showed that the risk and survival rates for epithelial OC were higher for white women, while African-American women had lower risk and survival rates compared to other racial/ethnic groups. Epidemiological studies suggest that some risk factor differences may exist between racial/ethnic groups, but the reasons for these differences are unknown.^[10]^ However, other studies have reported that the survival of OC patients is not influenced by race/ethnicity.^[16,17]^ In addition, a meta-analysis of 5-year survival rates for 106,704 U.S. OC patients showed no difference in survival between white and black women.^[18]^

**Figure 1.**
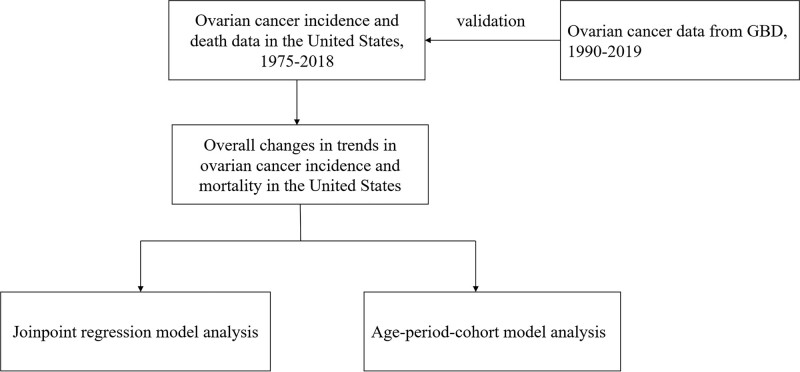
The flow chart of this study.

Considering the consistent decline in OC incidence and mortality, this study conducted a joinpoint regression model analysis and age-period-cohort model analysis based on the SEER database data. The joinpoint regression model was used to assess trends and transitions in OC incidence and incidence-based mortality rates (IBM) in the US population from 1975 to 2018, and the age-period-cohort model was used to determine the effects of age, period, and cohort factors on OC incidence and mortality. We also examined the impact of different races/ethnicities on the incidence and mortality of OC patients, which is essential to elucidate the risk profile of OC in the U.S. In addition, U.S. OC data from 1990 to 2019 obtained from the Global Burden of Disease (GBD) study can potentially serve as a validation tool to help explain some of the etiologic trends. Figure 1 shows the flow chart of this study.

## 2. Materials and Methods

### 2.1. Data collection

The National Cancer Institute (NCI) SEER program is the definitive source for cancer incidence and mortality data in the United States, including 22 regional cancer registries covering approximately 35% of the US population.^[19]^ We used the SEER*Stat statistical software (version 8.4.1.1) to obtain the required data. The incidence data were obtained from Incidence-SEER Research Data, 9 Registries, and Nov 2020 Sub (1975–2018). Mortality data were obtained from the Incidence-Based Mortality-SEER Research Data, 9 Registries, and Nov 2020 Sub (1975–2018). All OC cases were identified using the International Classification of oncology (ICD-O-3) code C56.9. To further analyze the trends in incidence and mortality rates among races in the US, we divided patients with OC into 4 groups: White, Black, and other. The “other” group includes American Indians/AK natives and Asian/Pacific islanders. All OC cases included in the study were actively followed-up, and cases with unknown details were excluded.

To further examine trends in morbidity and mortality in the U.S. population, we also analyzed the Global Burden of Disease Study (GBD 2019) data as a possible validation set. We collected population data from the Institute for Health Metrics and Evaluation (https://vizhub.healthdata.org/gbd-results/). on incident and mortality cases of OC in the U.S. from 1990 to 2019.

### 2.2. Incidence-based mortality

IBM incorporates mortality at the population level for specific tumor types such as OC in the SEER registry, linking OC mortality in the SEER registry to OC incidence (1975–2018). The numerator in the ratio is the number of OC-specific deaths reported to SEER. The denominator is the general population at-risk data obtained by the SEER program from the Bureau of the Census.^[20]^

### 2.3. Joinpoint regression analysis

This study was conducted using joinpoint regression software (version 4.9.0.1-February 2022) developed by the NCI.^[21]^ Kim et al^[22]^ proposed a joinpoint regression model in 2000, which uses the annual percent change (APC) and average annual percent change (AAPC) and their 95% confidence intervals (CIs) as indicators for analysis. Values of APC or AAPC greater than 0 (*P* < .05) or less than 0 (*P* < .05), or *P* > .05 represent an increase, a decrease, or no significant change in the index during the period, respectively.^[23]^ To analyze the trends and transitions in OC incidence and mortality from 1975 to 2018, we applied best-fit log-linear regression models to calculate APC, AAPC, and the corresponding 95% CIs and to determine the point of connection when a significant change in APC occurred (*P* < .05). In the SEER study, trends in OC incidence and IBM from 1975 to 2018 were fitted to a maximum of 2 joint points. In the GBD study, trends in OC incidence and mortality from 1990 to 2019 were fitted to a maximum of 2 joinpoints. To determine the trends of OC incidence and IBM among different races, we performed separate joinpoint analyses on OC patients of all races: Black, White, and others.

### 2.4. Age-period-cohort analysis

Age-period-cohort analysis can explore age, period, and cohort effects from observed age-specific mortality data, and can provide useful information for cancer incidence and mortality studies.^[24]^ This study used the NCI age-period-cohort analysis web tool (http://analysistools.nci.nih.gov/apc/) to assess the relationship between OC incidence and mortality in the U.S. and age, period, and cohort parameters.^[25]^ In this study, we arranged the data according to age, period, and cohort, with the same interval to fit the model conditions. The final age, period, and cohort were divided into 18 five-year age groups (0–4, 5–9, 10–14, ..., 85+), 9 periods (1975–1979, 1980–1984, ..., 2015–2018) and 26 cohorts (1890, 1895, 1900, ..., 2015). Net drift (%/year) was the most dominant result of the age-period-cohort model analysis and represented the overall temporal trend in mortality and incidence. This is similar to the annual percent change in mortality, but it considers both components of the trend attributable to period and the trend attributable to cohort factors.^[26]^ In addition, we calculated the rate ratio (RR) of morbidity or mortality for any given period or cohort adjusted for the effects of age and nonlinear cohort or period.

### 2.5. Statistical analysis

The incidence and IBM rates of OC were calculated according to year, age, and race using SEER*Stat statistical software (version 8.4.1.1). The incidence and IBM rates were age-adjusted to the standard U.S. population in 2000, and the values were expressed per 100,000 people per year. Joinpoint software (version 4.9.0.1) was used to analyze trends in the incidence and IBM of OC in the U.S. An age-period-cohort analysis model was used to assess the relationship between OC incidence and mortality in the U.S. with age, period, and cohort effects. For the GBD data, OC incidence and mortality rates were age-adjusted to the standard U.S. population in 2000. Statistical significance was set at *P* < .05.

## 3. Results

### 3.1. Overall changes in trends in OC incidence and mortality in the United States

**Figure 2.**
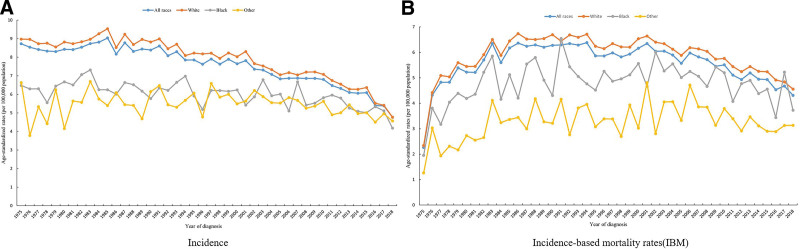
Trends in age-standardized rates of ovarian cancer (per 100,000 population) in the United States, 1975 to 2018. (A) Trends in ASIR. (B) Trends in ASMR. ASIR = age-standardized incidence rate, ASMR = age-standardized mortality rate, IBM = incidence-based mortality rates.

From 1975 to 2018, the incidence of OC in the U.S. population fluctuated, but the overall trend showed a decrease. The age-standardized incidence rate (ASIR) for the total population decreased from 8.73/100,000 population in 1975 to 4.77/100,000 population in Whites, and from 8.98/100,000 population in 1975 to 4.77/100,000 population in Blacks. The ASIR declined from 6.48/100,000 population in 1975 to 4.18/100,000 population, and the ASIR for other races declined from 6.63/100,000 population in 1975 to 4.56/100,000 population (Fig. 2A).

The U.S. age-standardized mortality rate (ASMR) for OC from 1975 to 2018 showed a fluctuating upward trend, rising from 2.27/100,000 population in 1975 to 4.31/100,000 population in the total population, with IBM reaching its peak in the U.S. population in 1994 with an ASMR of 6.38 per 100,000 population. The ASMR increased from 2.34/100,000 population in 1975 to 4.56/100,000 population for whites, from 1.95/100,000 population in 1975 to 3.73/100,000 population in blacks, and from 1.26/100,000 population in 1975 to 3.13/100,000 population in other races. The ASMR for other races increased from 1.26/100,000 people in 1975 to 3.13/100,000 population (Fig. 2B).

### 3.2. Joinpoint regression model analysis of OC incidence in the United States

**Figure 3.**
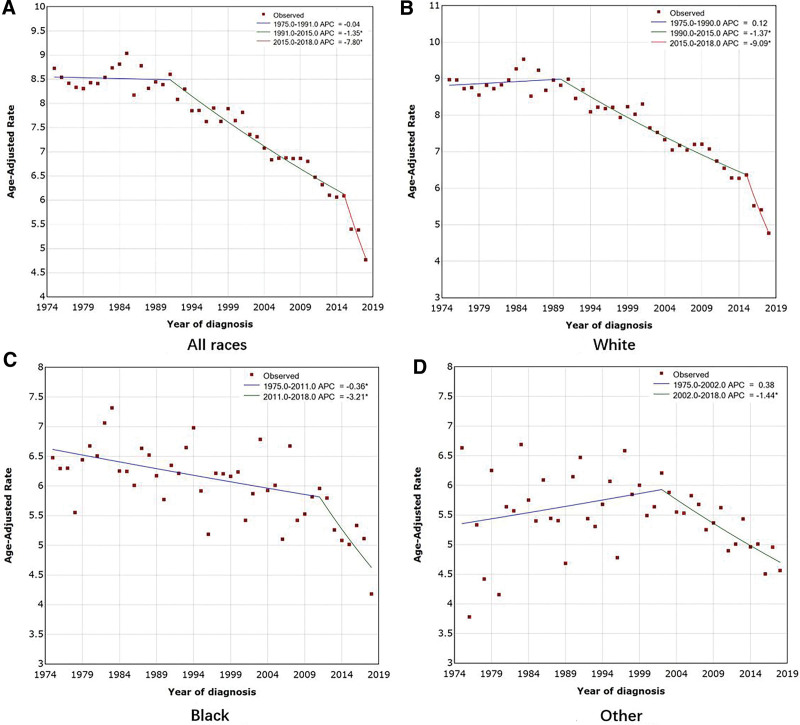
Trends in age-standardized incidence rates (per 100,000 population) of ovarian cancer in the United States from the SEER database, 1975 to 2018. (A) Trends in incident ovarian cancer incidence in all races. (B) Trends in incident ovarian cancer incidence in White populations. (C) Trends in incident ovarian cancer incidence in the black population. (D) Trends in incident ovarian cancer incidence in other races. *Indicates that APC is significantly different from zero at the α = 0.05 level. APC = annual percent change, SEER = Surveillance, Epidemiology, and End Results.

**Figure 4.**
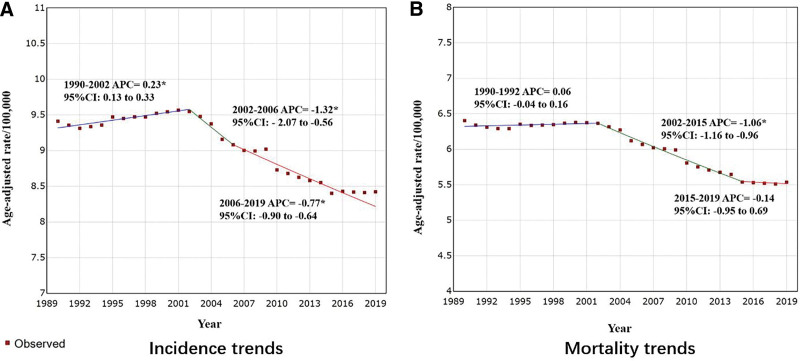
Trends in age-standardized rates (per 100,000 population) for ovarian cancer in the United States from the GBD database, 1990 to 2019. (A) Trends in incidence rates. (B) Trends in mortality rates. *Indicates that the APC is significantly different from zero at the α = 0.05 level. APC = annual percent change, GBD = Global Burden of Disease.

We used the joinpoint model to analyze the incidence of OC in the United States between 1975 and 2018 from data including 80,622 OC cases (Table 1). The trend in OC incidence is shown in Figure 2, and the AAPC is shown in Table 2. The incidence of OC showed a decreasing trend from year to year, when ethnic differences were not considered. The AAPC was −1.33% (95% CI: −1.64 to −1.02, *P* < .001). The incidence rate slowly decreased by 0.04% per year (95% CI: −0.37 to 0.29) from 1975 to 1991. From 1991 to 2015, the incidence rate decreased by 1.35% per year (95% CI: −1.52 to −1.18), and from to 2015–2018, it declined significantly by 7.80% per year (95% CI: −11.52 to −3.92) (Fig. 3A). A similar downward trend was observed with joinpoint analysis of OC incidence data from the GBD database from 1990 to 2019, with an AAPC of −0.43% (95% CI: −0.55 to −0.31, *P* < .001) (Tables 3 and 4, Fig. 4A).

**Table 1:** Joinpoint trends of ovarian cancer incidence in the SEER database from 1975 to 2018 (n = 80,622).

Racial	Trend 1			Trend 2			Trend 3		
	Years	APC (95% CI)	*P*	Years	APC (95% CI)	*P*	Years	APC (95% CI)	*P*
All races	1975–1991	−0.04 (−0.37 to 0.29)	.800	1991–2015	−1.35* (−1.52 to −1.18)	<.001	2015–2018	−7.80* (−11.52 to −3.92)	<.001
White	1975–1999	0.12 (−0.27 to 0.52)	.522	1990–2015	−1.37* (−1.55 to −1.19)	<.001	2015–2018	−9.09* (−13.41 to −4.54)	<.001
Black	1975–2011	−0.36* (−0.62 to −0.10)	.008	2011–2018	−3.21* (−5.54 to −0.83)	.010	–	–	–
Other	1975–2002	0.38 (−0.15 to 0.91)	.155	2002–2018	−1.44* (−2.17 to −0.70)	<.001	–	–	–

APC = annual percent change, CI = confidence interval, SEER = Surveillance, Epidemiology, and End Results.

* Indicates that APC is significantly different from zero at the α = 0.05 level.

**Table 2:** AAAPC of ovarian cancer incidence in the United States from 1975 to 2018.

Racial	All races	White	Black	Other
AAPC	−1.33*	−1.41*	−0.83*	−0.30
95% CI	−1.64 to −1.02	−1.78 to −1.05	−1.26 to −0.39	−0.72 to 0.12
*P*	<.001	<.001	<.001	.156

AAPC = average annual percent change, CI = confidence interval.

* Indicates that APC is significantly different from zero at the α = 0.05 level.

**Table 3:** APC in incidence and mortality of ovarian cancer in the United States from the GBD database, 1990 to 2019.

Racial	Trend 1			Trend 2			Trend 3		
	Years	APC (95% CI)	*P*	Years	APC (95% CI)	*P*	Years	APC (95% CI)	*P*
Incidence	1990–2002	0.23* (0.13 to 0.33)	<.001	2002–2006	−1.32* (−2.07 to −0.56)	.002	2006–2019	−0.77* (−0.90 to −0.64)	<.001
Mortality	1990–2002	0.06 (−0.04 to 0.16)	.233	2002–2015	−1.06* (−1.16 to −0.96)	<.001	2015–2019	−0.14 (−0.95 to 0.69)	.7733

APC = annual percent change, CI = confidence interval, GBD = Global Burden of Disease.

* Indicates that APC is significantly different from zero at the α = 0.05 level.

**Table 4:** AAPC in ovarian cancer incidence and mortality in the United States from the GBD Database, 1990 to 2019.

	AAPC	95% CI	*P*
Incidence	−0.43*	−0.55 to −0.31	<.001
Mortality	−0.47*	−0.59 to −0.35	<.001

AAPC = average annual percent change, CI = confidence interval, GBD = Global Burden of Disease.

* Indicates that APC is significantly different from zero at the α = 0.05 level.

We followed this investigation with a racial/ethnicity comparison and noted a slow increase and then a significant decrease in the incidence of OC in Caucasians: AAPC = −1.41% (95% CI: −1.78 to −1.05, *P* < .001). The incidence increased slowly at a rate of 0.12% per year (95% CI: −0.27 to 0.52) from 1975 to 1999, decreased at a rate of 1.37% per year from 1990 to 2015, and then decreased significantly at a rate of 9.09% per year (95% CI: −13.41 to −4.54) from 2015 to 2018 (Fig. 3B). OC incidence for blacks declined slowly at a rate of 0.36% per year (95% CI: −0.62 to −0.10) from 1975 to 2011 and then declined significantly at 3.21% per year (95% CI: −5.54 to −0.83) from 2011 to 2018 (Fig. 3C). The trend of the incidence of OC in “others” showed an inverted “V” curve. From 1975 to 2002, the incidence increased slowly at a rate of 0.38% (95% CI: −0.15 to 0.91) and then decreased significantly at an annual rate of 1.44% (95% CI: −2.17 to −0.70) from 2002 to 2018 (Fig. 3D).

### 3.3. Joinpoint regression model analysis of incidence-based mortality rates from OC in the United States

**Figure 5.**
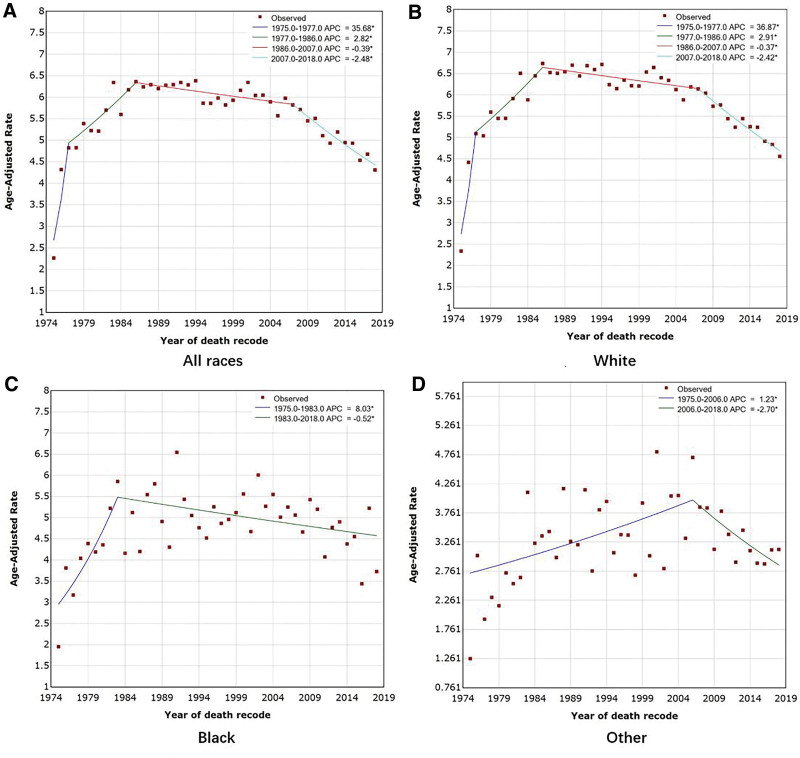
Trends in age-standardized OC rates (per 100,000 population) in the United States, 1975 to 2018, from the SEER database. (A) Trends in IBM of OC across all races. (B) IBM trends in White OC. (C) IBM trends in Black OC. (D) IBM trends in OC of other races. *Indicates that APC is significantly different from zero at the α = 0.05 level. APC = annual percent change, IBM = incidence-based mortality, OC = ovarian cancer.

Throughout the study period (1975–2018), 60,218 OC-related deaths occurred (Table 5). The OC mortality trend is shown in Figure 5, and the AAPC is shown in Table 6. OC mortality in the U.S. population showed an inverted “V” pattern, with an increase followed by an immediate decrease. AAPC was −1.17% (95% CI: 0.32 to 2.03, *P* = .007), and the mortality rate was 35.68% (95% CI: 14.14 to 61.28) per year from 1977 to 1986, continuing to increase at a rapid rate of 2.82% (95% CI: 1.49 to 4.16) per year from 1991 to 2015. Mortality peaked in 1986, with an observed age-adjusted mortality rate of 6.36%/100,000 population and a modeled age-adjusted mortality rate of 6.34%/100,000 population. Subsequently, mortality declined at a rate of 0.39% (95% CI: −0.66 to −0.12) per year from 1986 to 2007 and continued to decline significantly at a rate of 2.48% (95% CI: −3.09 to −1.85) per year from 2007 to 2018 (Fig. 5A). A joinpoint analysis of OC mortality data from the GBD database for 1990 to 2019 showed a similar sharp increase followed by a decrease, with an AAPC as −0.47% (95%CI: −0.59 to −0.35, *P* < .001) (Tables 3 and 4, Fig. 4B).

**Table 5:** Joinpoint trends of IBM rates from ovarian cancer in the SEER database, 1975 to 2018 (n = 60,218).

Race	Trend 1			Trend 2			Trend 3			Trend 4		
	Years	APC (95% CI)	*P*	Years	APC (95% CI)	*P*	Years	APC (95% CI)	*P*	Years	APC (95% CI)	*P*
All races	1975–1977	35.68* (14.14 to 61.28)	.001	1977–1986	2.82* (1.49 to 4.16)	<.001	1986–2007	−0.39* (−0.66 to −0.12)	.006	2007–2018	−2.48* (−3.09 to −1.85)	<.001
White	1975–1977	36.87* (17.66 to 59.21)	<.001	1977–1986	2.91* (1.73 to 4.09)	<.001	1986–2007	−0.37* (−0.61 to −0.13)	.004	2007–2018	−2.42* (−2.99 to −1.85)	<.001
Black	1975–1983	8.03* (1.51 to 14.97)	.016	1983–2018	−0.52* (−0.93 to −0.10)	.016	—	—	—	—	—	—
Other	1975–2006	1.23* (0.45 to 2.01)	.003	2002–2018	−2.70* (−4.49 to −0.87)	.005	—	—	—	—	—	—

APC = annual percent change, CI = confidence interval, IBM = incidence-based mortality, SEER = Surveillance, Epidemiology, and End Results.

* Indicates that APC is significantly different from zero at the α = 0.05 level.

**Table 6:** AAPC of IBM of ovarian cancer in the United States from the SEER database, 1975 to 2018.

Race	All races	White	Black	Other
AAPC	−1.17*	−1.26*	1.02	0.12
95% CI	0.32 to 2.03	0.51 to 2.01	−0.15 to 2.21	−0.62 to 0.86
*P*	.007	.001	.088	.760

AAPC = average annual percent change, CI = confidence interval, IBM = incidence-based mortality, SEER = Surveillance, Epidemiology, and End Results.

* Indicates that APC is significantly different from zero at the α = 0.05 level.

White OC mortality also followed an inverted V pattern, with an increasing trend immediately followed by a decreasing trend (AAPC = −1.26%, 95% CI: 0.51 to 2.01, and *P* = .001) (Fig. 5B). Mortality from OC in Blacks from 1975 to 1983 increased significantly at an annual rate of 8.03% (95% CI: 1.51 to 14.97), peaking in 1983 with an observed age-adjusted mortality rate of 5.86%/100,000 population and a modeled age-adjusted mortality rate of 5.48%/100,000 population. Subsequently, it declined slowly at a rate of 0.52% (95% CI: −0.93 to −0.10) per year from 1983 to 2018 (Fig. 5C). Other OCs showed a similar upward and then downward trend, peaking later in 2006, with an observed age-adjusted mortality rate of 4.71%/100,000 population and a model age-adjusted mortality rate of 3.98%/100,000 population (Fig. 5D).

### 3.4. Age-period-cohort model analysis of OC incidence and IBM in the United States

Table S1, Supplemental Digital Content, http://links.lww.com/MD/K898 shows a net drift of −0.83% (95% CI: −1.02 to −0.64) for the U.S. population prevalence and −1.00% (95% CI: −1.62 to −0.37) for IBM, indicating a decrease in prevalence and IBM. In addition, we noted that the local drift was different at different ages: taking 72.5 years of age as the boundary, the mortality rate below 72.5 years decreased year by year, but the mortality rate above 72.5 years increased year by year (Figure S1, Supplemental Digital Content, http://links.lww.com/MD/K897).

**Figure 6.**
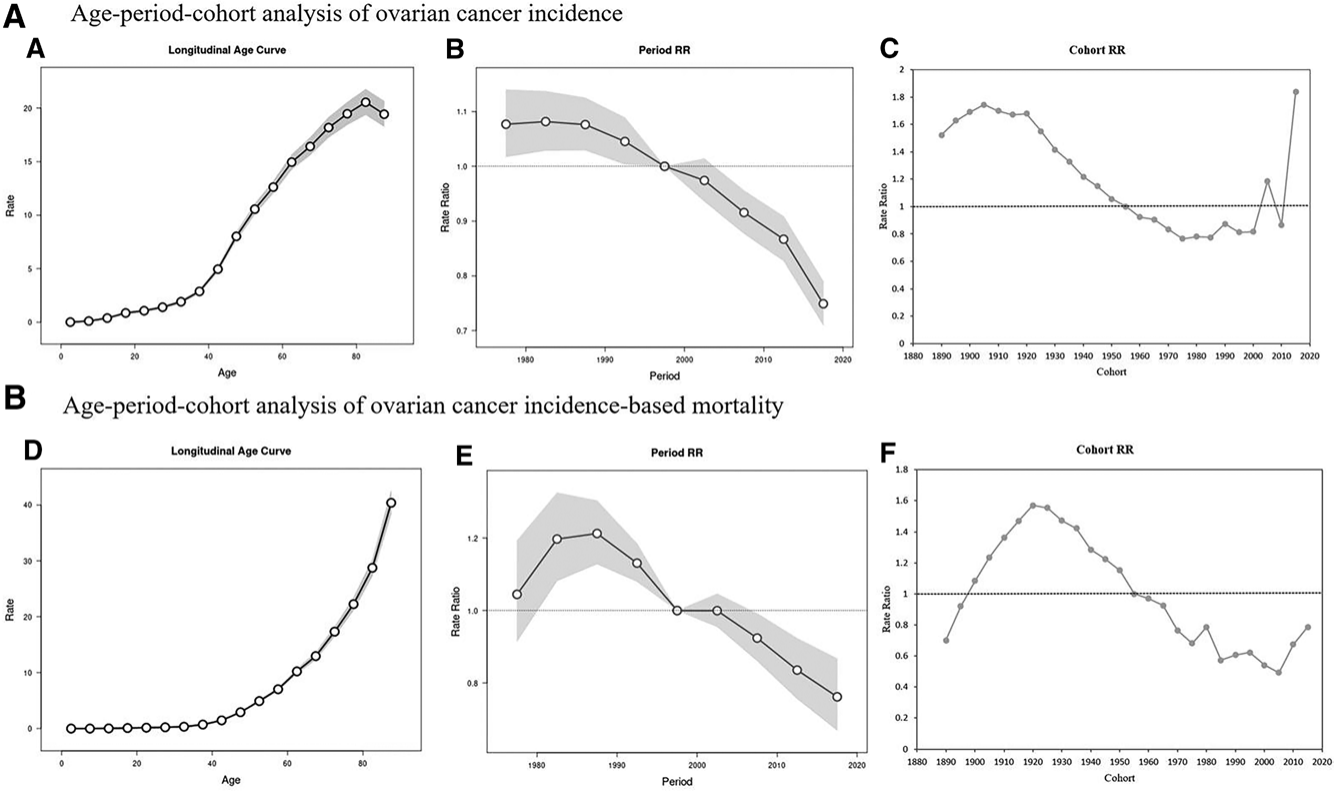
An age-period-cohort model analysis of ovarian cancer incidence and incidence-based mortality in the United States, SEER database, 1975 to 2018. The gray zone indicates 95% CIs. A. The age effect analyzed (A), the period rate ratio (B), and the cohort rate ratio (C) of OC incidence. B. The age effect analyzed (D), the period rate ratio (E), and the cohort rate ratio (F) of OC incidence-based mortality. OC = ovarian cancer, RR = rate ratio, SEER = Surveillance, Epidemiology, and End Results.

Figure 6 shows the age-period-cohort analysis associated with the number of OC patients and IBM in the U.S. population. OC morbidity and mortality rates increased with age (Fig. 6A and D; Table S2, Supplemental Digital Content, http://links.lww.com/MD/K899). In the same birth cohort, its prevalence increased with age, rising rapidly after the age of 40 years, and finally reaching its highest point in the 80 to 84 years age group (20.54%). The mortality rate showed a similar trend of gradual increase with age, reaching its highest rate (40.39%) in the ≥85 years age group.

In 1995 to 1999, there was a significant effect of period on OC morbidity and mortality (Fig. 6B and E; Table S3, Supplemental Digital Content, http://links.lww.com/MD/K900), with an increasing trend in the RR of morbidity and mortality (*P* < .001) from 1980 to 1984 and 1995 to 1999, while in the period from 2005 to 2009, the RR of morbidity was 0.92 (95% CI: 0.88 to 0.96) and the RR for mortality was 0.92 (95% CI: 0.86 to 0.99), decreasing from the reference period 1995 to 1999. The trend in RR suggests a phase-in effect of reducing the incidence of OC, and the risk of OC-related mortality occurred from 1995 to 1999, which led to a reduction in incidence and mortality risk from 2005 to 2009. Of note, a phase effect of reducing the occurrence of OC was observed in the GBD data during 2002–2006 (APC = −1.32%; 95% CI: −2.07 to −0.56; *P* = .002), and a phase effect of reducing the risk of OC-related mortality was observed during 2002–2005 (APC = −1.06%. 95% CI: −1.16 to −0.96; *P* < .001) (Fig. 4).

Figure 6C and F further illustrate the cohort effect analysis for incidence and mortality, respectively. The RR of OC incidence showed a decreasing and then an increasing trend. The highest RR was observed in patients born between 1905 and 1909 (RR = 1.74; 95% CI: 1.62 to 1.87), which we used as the reference birth cohort, suggesting that they had the highest risk of developing OC among all birth cohorts. The lowest RR (RR = 0.77; 95% CI: 0.69 to 0.85) was observed during 1975–1979, suggesting that an effect of reducing OC incidence occurred during 1910 to 1974. The highest RR (RR = 1.84; 95% CI: 0.22 to 15.69) was reached again in 2015 to 2019, suggesting that an effect of increasing OC incidence occurred during 2015 to 2019. However, the cohort effect was different between incidence and mortality, with the OC IBM reaching its highest RR (RR = 1.57; 95% CI: 1.49 to 1.65) in 1920–1924, which was comparable to the peak RR for OC occurring in births from 1905 to 1909. This was followed by the lowest RR (RR = 0.49; 95% CI: 0.07 to 3.25) in 2005–2009 (Table S3, Supplemental Digital Content, http://links.lww.com/MD/K900). In contrast, in the GBD cohort, there was a steady but uneven reduction in the risk of OC occurrence and associated death (Fig. 4).

## 4. Discussion

OC is the leading cause of death among gynecological cancer patients in the United States.^[27]^ Studies have shown that there are differences in survival rates among patients of different races, even when the same treatment regimens are used.^[28]^ This study assessed the long-term trends in OC incidence and mortality in the United States from 1975 to 2018 based on the SEER database, with an AAPC of −1.33% (95% CI: −1.64 to −1.02) and a net drift of −0.83% (95% CI: −1.02 to −0.64) per year for incidence and an AAPC of −1.17% for mortality (95% CI: 0.32 to 2.03), with a net drift of −1.00% per year (95% CI: −1.62 to −0.37). We reported a significant decline in the incidence of OC in the U.S. population and a continued decline in IBM. White OC mortality rates have declined significantly since 1986, Black OC mortality rates have declined since 1983, and OC mortality rates in other races have declined since 2002. A decline in OC incidence and mortality cannot be achieved without efforts in prevention, diagnosis, and treatment.

Our study reported significant reductions in OC incidence and IBM in the US population. Our findings are similar to those of Park et al,^[7]^ who analyzed trends by race/ethnicity in OC data from to 2000–2013 in the SEER-18 database. The difference is that we examined trends over a longer period and used more recent data (1975–2018). Sopik et al^[29]^ also reported that OC mortality in the U.S. decreased by 8% and 18% during 1975–1991 and 1992–2011, respectively. Our study reported a decline in OC mortality since 1986, with APC of −0.39% (95% CI: −0.66 to −0.12) from 1986 to 2007 and APC of −2.48% (95% CI: −3.09 to −1.85) from 2007 to 2018. A study by Phung et al^[9]^ found that the incidence of high-grade plasmacytoid carcinoma, the most common histologic subtype of OC, declined significantly across all racial/ethnic groups during 1992–2019, notably with the greatest decline in white women from to 2010–2019 (AAPC = −6.1%; 95% CI: −8.0 to −4.2). Consistent with the report by Phung et al, our study showed a significant decline in OC prevalence across all races since 1991, with the greatest decline in white women from 2015 to 2018 (APC = −9.09%; 95% CI: −13.41 to −4.54). The above studies, although conducted using different time points, demonstrated reductions in OC incidence and mortality in the U.S. population.

The underlying reasons for the decline in OC incidence and mortality across all racial and ethnic populations are complex, multifaceted, and not fully understood. One study found that increased duration of oral contraceptive use, increased number of pregnancies, breastfeeding, and a history of tubal ligation or hysterectomy with preservation of the ovaries were associated with a reduced risk of OC in the United States, with significant and long-lasting effects on incidence and mortality.^[30]^ Interestingly, the probability of developing OC depends on the cumulative effect of the 4 risk factors mentioned above, with the estimated risk of OC for women exposed to the 4 risk factors being approximately 0.35%, whereas the lifetime risk of OC in the absence of exposure to the 4 risk factors was estimated to be approximately 2.8%.^[31]^ The oral contraceptive pill was first introduced in the United States in 1960.^[32]^ Women who had used oral contraceptives had an average 25% lower risk of OC than those who had never used them, and the level of protection was proportional to the duration of use, with significant reductions in risk more than 30 years after stopping the use of the pill.^[33]^ A study by Sopik et al^[6]^ found that between 1990 and 2015, the percentage of women over the age of 70 who had taken oral contraceptives increased from approximately 20% to 85%. This is consistent with our finding that the incidence rate declined by 1.35% per year from 1991 to 2015 (95% CI: −1.52 to −1.18). The proportion of breastfeeding mothers began to increase in 1975 and stabilized at 70–75% among mothers born in 1960 or later. The increase in the proportion of breastfeeding mothers is attributable to the increased awareness of the benefits of breastfeeding and efforts to raise breastfeeding awareness.^[34]^ In addition, an increase in salpingectomy may be a possible factor in the declining incidence of OC. Women born between 1940 and 1949 who had a salpingectomy had a 5% lower risk of OC than women who did not undergo salpingectomy.^[6]^ This may be the reason for the lowest risk for OC incidence in 1975–1979 (RR = 0.77; 95% CI: 0.69 to 0.85).

Cisplatin and carboplatin were approved for OC treatment in the U.S. in 1978 and 1989, respectively.^[29]^ Ozols et al^[35]^ reported a 16% reduction in the risk of death in the group receiving carboplatin combined with paclitaxel compared with the cisplatin plus paclitaxel group, suggesting a slight increase in the efficacy of carboplatin. In a study by Neijt et al in 1984, 186 patients with advanced epithelial OC were treated with a combination of hexamethyltrimethoprim, cyclophosphamide, methotrexate, and 5-fluorouracil (Hexa-CAF), or a combination of cyclophosphamide, hexamethyltrimethoprim, adriamycin, and cisplatin (CHAP-5). The CHAP-5 regimen showed better overall remission (*P* = .0001) and longer survival (*P* < .002), suggesting that the CHAP-5 regimen is one of the most effective treatments for OC.^[36]^ In 1992, paclitaxel was approved as the first-line treatment for advanced OC.^[29]^ The introduction of paclitaxel increased the remission rate from 60% to approximately 75%; however, the relapse rate remained high with the addition of paclitaxel (approximately 87%) compared with the cisplatin alone group (10-year relapse rate of 90%).^[29,37]^ In the SEER database, after the introduction and expanded use of paclitaxel, the 5- and 12-year mortality rates for patients with advanced OC decreased by approximately 6% and 2%, respectively.^[29]^ In contrast, Sopik et al found that the introduction of taxanes, targeted drugs, and surgery did not improve the cure rate of OC. The decline in OC mortality and the resultant decrease in incidence are largely attributable to the introduction of oral contraceptives in 1960 and their expanded use during the period 1960–1990. However, OC mortality did not decline at the onset, suggesting that early detection did not lead to a reduction in mortality rates.^[29]^ This is consistent with our findings that morbidity began to decline in 1975 and mortality has declined since 1986, with APC = −0.39% (95% CI: −0.66 to −0.12) from 1986 to 2007 and APC = −2.48% (95% CI: −3.09 to −1.85) from 2007 to 2018.

The age effect is the risk of morbidity or mortality due to age-related factors and the impact of changes in an individual’s physiological state as they grow older.^[38]^ Age has long been recognized as an independent poor prognostic factor for OC and is associated with poor management and treatment interruption.^[39]^ The results of this study showed that the risk of OC morbidity and mortality increased with age, with the highest risk of morbidity in the age group of 80 to 84 years and the highest risk of mortality in the ≥85 years age group. The study demonstrated that more than 50% of OC patients are over the age of 65 years, and with the aging of the population, the number of older women with OC is increasing. In addition, the anatomical location of OC is in the pelvic cavity, with insidious onset and long latency period, which makes it difficult to detect in the early stages of the disease, and the age at diagnosis increases accordingly, and older women are more likely to be initially diagnosed with advanced disease.^[40–42]^ Older patients, especially those aged over 80 years, are unlikely to undergo surgery to achieve optimal cytoreduction, and there is significant toxicity associated with aggressive primary surgical cytoreduction.^[40]^ Moore et al^[43]^ conducted a retrospective study of 85 patients aged >80 years who underwent cytopaenic surgery and showed that 13% died before discharge and 20% died within 60 days of surgery. Prognostic problems in older women with OC require attention, and a deeper understanding of the biological differences between younger and older patients and improving the tolerability of treatments received by older women will help improve the problem of poor prognosis in older women.

The results of the period effect showed an overall decreasing trend in the risk of morbidity and mortality from OC as the period increased, with the highest risk of mortality in 1985–1989. Wang et al^[44]^ showed that dietary patterns are an important factor for cancer morbidity and mortality, and it was shown that a healthy dietary pattern reduced the risk of OC by 14%, while a high intake of both red meat and processed meat increased the risk of OC by 19%. Red meat intake in the U.S. declined significantly during 1990–2010 (−4.7 g/d).^[45]^ The correlation between smoking and the risk of OC should not be ignored, as it has been shown that women who have ever smoked have a 6% higher risk of developing OC than women who have never smoked.^[46]^ From 1980 to 2012, the global female smoking prevalence declined from 10.6% to 6.2%, a decrease of 1.7% per year, while in 2012, the daily rate of female smoking in the U.S. was less than 15%.^[47]^ Oral contraceptives (OCPs) are recognized as protective agents against OC, with an overall protection rate of approximately 40% among all OCPs users, which increases with the duration of their use. Compared to Asian and Central and Eastern European countries, the use of OCPs in the U.S. is as high as 16%.^[48]^ These factors may contribute to the decreased risk of developing and dying from OC.

Cohort effect is the risk of morbidity/mortality caused by exposure to a factor at a different age for people in the same birth cohort or at the same age for people in different cohorts.^[49]^ The results of the cohort effect showed that the later the birth cohort, the lower the risk of morbidity; however, the risk of morbidity for the 2015 birth cohort was at its maximum (RR = 1.84). The increased risk of incidence in 2015 may be due to the increased social pressure they are facing, and chronic stress can weaken the original anti-tumor environment of immune cells and promote tumor growth, invasion and metastasis, thus increasing the morbidity of OC.^[50]^ A study reported that hormone replacement therapy (HRT) increased the risk of OC by 37% compared to women who had never used HRT before.^[51]^ In the United States, the prevalence of HRT increased dramatically until 2002, but the prevalence of HRT was halved in 2002 because of the increased risk of OC associated with its use.^[51,52]^ All of these findings may favor a decrease in the risk of developing OC. The risk of death progressively decreased with cohort effects, with the risk of death decreasing later at birth. This may be related to the emergence of various therapeutic modalities and improved surgical protocols that allow for timely treatment of the disease. Reducing the healthcare burden of OC patients and enabling timely and effective treatment will reduce the risk of death in OC patients.

However, this study has some limitations. First, the SEER database lacks information on the etiological risk factors. Therefore, we collected data from the GBD as a possible validation set to complement the results of the SEER database. Secondly, given that GBD 2019 is an estimate of the burden of disease using a variety of mathematical models and is not a realistic observation, while GBD 2019 uses a variety of strategies to improve the quality and comparability of the data, errors from the actual situation are unavoidable. In addition, GBD lacks data on detailed histopathologic typing, staging, and treatment modalities for OC, and it would be meaningful to study the impact of different pathologic types, staging, and treatment modalities on the disease burden of OC. Finally, this study was only an attempt to formulate a scientific hypothesis on the causal relationship between OC morbidity and mortality trends based on available data and related literature. Nonetheless, the SEER database provides representative data for approximately 30% of the U.S. population,^[31]^ and GBD 2019 provides disease burden data for 369 diseases and injuries and 87 risk factors for 204 countries and territories, with disease burden metrics such as morbidity, prevalence, mortality, and disability-adjusted life years,^[53]^ which can provide a sufficiently large amount of data to help us analyze the trends in OC morbidity and mortality in the U.S. population.

## 5. Conclusions

In summary, this study reports a significant decline in OC morbidity and mortality in the U.S. since 1986. We examined the effects of age, period, and birth cohort on changes in trends using an age-period-cohort model. In addition, this study analyzed changes in trends in OC incidence and mortality by race/ethnicity in the United States. Differences in incidence trends between races/ethnicities may be due to differences in the prevalence of risk factors across years and birth cohorts. Reducing the disease burden and ethnic/racial disparities in OC may benefit from the implementation of effective preventive measures. Simultaneously, monitoring the trends in OC incidence and mortality by race/ethnicity will facilitate the development of targeted prevention and treatment measures.

## Acknowledgments

We thank the Institute for Health Metrics and Evaluation for the Development of the GBD database. We thank the National Cancer Institute and the SEER staff for providing this invaluable database.

## Author contributions

**Data curation:** Jiahui He.

**Formal analysis:** Jiahui He.

**Funding acquisition:** Qinyong Hu.

**Resources:** Qinyong Hu.

**Software:** Jiahui He.

**Writing – original draft:** Jiahui He.

**Writing – review & editing:** Qinyong Hu.

## Supplementary Material








