# Supplementary material for: Ovarian cancer disease burden decreased in the United States from 1975 to 2018: A joinpoint and age-period-cohort analysis
Source: Medicine (Baltimore). 2023 Dec 1;102(48):e36029. doi: 10.1097/MD.0000000000036029 (PMC10695534; doi:10.1097/MD.0000000000036029)
Supplement: Supplementary file 1 [file medi-102-e36029-s001.docx]

| Age | Incidence (95%CI) | | IBM (95%CI) | |
| --- | --- | --- | --- | --- |
|  | Percent per Year | 95%CI | Percent per Year | 95%CI |
| 2.5 | 1.5448 | -1.6497 to 4.8429 | -0.1055 | -10.1158 to 11.0196 |
| 7.5 | 0.5327 | -0.9731 to 2.0615 | -0.7154 | -7.1543 to 6.1701 |
| 12.5 | 0.4156 | -0.3109 to 1.1475 | -1.3286 | -4.0278 to 1.4464 |
| 17.5 | -0.238 | -0.7617 to 0.2884 | -1.3781 | -2.8629 to 0.1293 |
| 22.5 | -0.4554 | -0.8479 to -0.0613 | -1.3985 | -2.3257 to -0.4625 |
| 27.5 | -0.6704 | -0.9817 to -0.3581 | -1.6373 | -2.3075 to -0.9625 |
| 32.5 | -1.0312 | -1.2954 to -0.7663 | -1.7118 | -2.2213 to -1.1996 |
| 37.5 | -1.1822 | -1.401 to -0.963 | -1.5264 | -1.8835 to -1.168 |
| 42.5 | -1.3103 | -1.4913 to -1.129 | -1.7271 | -1.9894 to -1.464 |
| 47.5 | -1.3118 | -1.457 to -1.1663 | -1.5458 | -1.7284 to -1.3628 |
| 52.5 | -1.3732 | -1.4957 to -1.2506 | -1.3708 | -1.5065 to -1.235 |
| 57.5 | -1.4698 | -1.5798 to -1.3597 | -1.2883 | -1.3996 to -1.1769 |
| 62.5 | -1.3895 | -1.4953 to -1.2837 | -1.0212 | -1.1197 to -0.9225 |
| 67.5 | -1.2655 | -1.3745 to -1.1563 | -0.5667 | -0.6597 to -0.4735 |
| 72.5 | -1.0913 | -1.2113 to -0.9712 | -0.1117 | -0.2072 to -0.0162 |
| 77.5 | -0.8505 | -0.9919 to -0.7089 | 0.4398 | 0.3335 to 0.5461 |
| 82.5 | -0.5281 | -0.7089 to -0.347 | 1.0932 | 0.9606 to 1.226 |
| 87.5 | -0.1642 | -0.4398 to 0.1121 | 1.8339 | 1.6229 to 2.0452 |

Table S1. Incidence and the IBM local Drifts with net drift.

Abbreviations: IBM=incidence-based mortality.
