# Supplementary figures and images for: Ovarian cancer disease burden decreased in the United States from 1975 to 2018: A joinpoint and age-period-cohort analysis
Source: Medicine (Baltimore). 2023 Dec 1;102(48):e36029. doi: 10.1097/MD.0000000000036029 (PMC10695534; doi:10.1097/MD.0000000000036029)

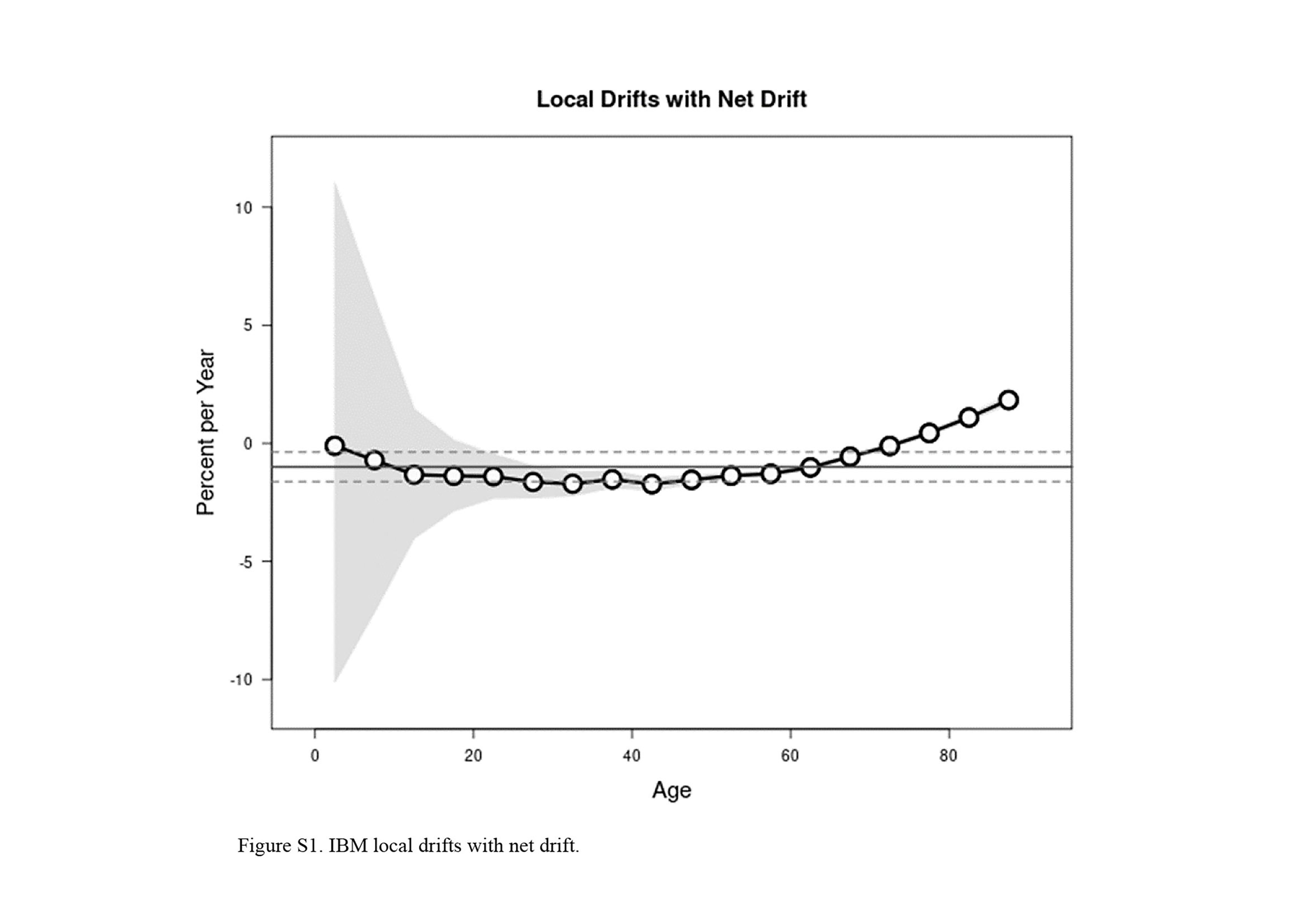

Supplement: Supplementary file 2 [file medi-102-e36029-s002.jpg]
