# Supplementary material for: Ovarian cancer disease burden decreased in the United States from 1975 to 2018: A joinpoint and age-period-cohort analysis
Source: Medicine (Baltimore). 2023 Dec 1;102(48):e36029. doi: 10.1097/MD.0000000000036029 (PMC10695534; doi:10.1097/MD.0000000000036029)
Supplement: Supplementary file 3 [file medi-102-e36029-s003.docx]

Table S2. Incidence and the IBM longitudinal age curve.

| Age(years) | Incidence (95%CI) | | | IBM (95%CI) | | |
| --- | --- | --- | --- | --- | --- | --- |
|  | Rate | CILo | CIHi | Rate | CILo | CIHi |
| 00-04 | 0.0157 | 0.0059 | 0.0421 | 0.0018 | 0.0002 | 0.018 |
| 05-09 | 0.112 | 0.0789 | 0.1588 | 0.0018 | 0.0002 | 0.0157 |
| 10-14 | 0.3893 | 0.3205 | 0.4728 | 0.0286 | 0.0158 | 0.0517 |
| 15-19 | 0.8659 | 0.7585 | 0.9884 | 0.0955 | 0.0702 | 0.1299 |
| 20-24 | 1.0782 | 0.964 | 1.206 | 0.1593 | 0.1279 | 0.1984 |
| 25-29 | 1.4054 | 1.2798 | 1.5432 | 0.2279 | 0.1917 | 0.271 |
| 30-34 | 1.9152 | 1.7677 | 2.0749 | 0.3321 | 0.2906 | 0.3794 |
| 35-39 | 2.8837 | 2.6969 | 3.0834 | 0.7167 | 0.6536 | 0.786 |
| 40-44 | 4.9573 | 4.6865 | 5.2437 | 1.4693 | 1.3704 | 1.5752 |
| 45-49 | 8.0221 | 7.6424 | 8.4206 | 2.9246 | 2.7676 | 3.0905 |
| 50-54 | 10.5615 | 10.0905 | 11.0544 | 4.9173 | 4.6826 | 5.1636 |
| 55-59 | 12.6203 | 12.0682 | 13.1976 | 7.0369 | 6.7186 | 7.3703 |
| 60-64 | 14.955 | 14.2821 | 15.6595 | 10.2268 | 9.7669 | 10.7084 |
| 65-69 | 16.419 | 15.6192 | 17.2598 | 12.9643 | 12.3386 | 13.6218 |
| 70-74 | 18.1823 | 17.2687 | 19.1442 | 17.3413 | 16.4994 | 18.2261 |
| 75-79 | 19.4718 | 18.4487 | 20.5517 | 22.2619 | 21.1658 | 23.4148 |
| 80-84 | 20.5447 | 19.3908 | 21.7674 | 28.7564 | 27.3077 | 30.2821 |
| 85+ | 19.4295 | 18.2832 | 20.6478 | 40.3888 | 38.3708 | 42.5129 |

Abbreviations: IBM=incidence-based mortality.
