# Supplementary material for: Ovarian cancer disease burden decreased in the United States from 1975 to 2018: A joinpoint and age-period-cohort analysis
Source: Medicine (Baltimore). 2023 Dec 1;102(48):e36029. doi: 10.1097/MD.0000000000036029 (PMC10695534; doi:10.1097/MD.0000000000036029)
Supplement: Supplementary file 4 [file medi-102-e36029-s004.docx]

Table S3. Incidence and IBM RR values and 95%CI of age, period and cohort.

|  | Incidence (95%) | | | IBM (95%) | | |
| --- | --- | --- | --- | --- | --- | --- |
|  | Rate Ratio | CILo | CIHi | Rate Ratio | CILo | CIHi |
| Age |  |  |  |  |  |  |
| 2.5 | 1.2529 | 1.1106 | 1.4135 | 1.5371 | 1.0649 | 2.2187 |
| 7.5 | 1.2016 | 1.0745 | 1.3437 | 1.462 | 1.045 | 2.0453 |
| 12.5 | 1.1524 | 1.0395 | 1.2775 | 1.3905 | 1.0254 | 1.8856 |
| 17.5 | 1.1052 | 1.0055 | 1.2148 | 1.3225 | 1.0061 | 1.7385 |
| 22.5 | 1.0599 | 0.9723 | 1.1555 | 1.2579 | 0.9871 | 1.603 |
| 27.5 | 1.0165 | 0.9399 | 1.0994 | 1.1964 | 0.9682 | 1.4784 |
| 32.5 | 0.9749 | 0.9082 | 1.0465 | 1.1379 | 0.9495 | 1.3638 |
| 37.5 | 0.9350 | 0.8771 | 0.9967 | 1.0823 | 0.9308 | 1.2585 |
| 42.5 | 0.8967 | 0.8464 | 0.9500 | 1.0294 | 0.9118 | 1.1622 |
| 47.5 | 0.8600 | 0.8159 | 0.9065 | 0.9791 | 0.8920 | 1.0747 |
| 52.5 | 0.8248 | 0.7854 | 0.8661 | 0.9312 | 0.8700 | 0.9967 |
| 57.5 | 0.7910 | 0.7547 | 0.829 | 0.8857 | 0.842 | 0.9317 |
| 62.5 | 0.7586 | 0.7239 | 0.795 | 0.8424 | 0.8014 | 0.8855 |
| 67.5 | 0.7275 | 0.6929 | 0.7639 | 0.8012 | 0.7498 | 0.8562 |
| 72.5 | 0.6977 | 0.6621 | 0.7353 | 0.7621 | 0.6957 | 0.8348 |
| 77.5 | 0.6692 | 0.6318 | 0.7087 | 0.7248 | 0.6434 | 0.8166 |
| 82.5 | 0.6418 | 0.6022 | 0.6839 | 0.6894 | 0.5942 | 0.7999 |
| 87.5 | 0.6155 | 0.5736 | 0.6604 | 0.6557 | 0.5483 | 0.7841 |
| Period |  |  |  |  |  |  |
| 1975-1979 | 1.0768 | 1.0173 | 1.1398 | 1.0447 | 0.9152 | 1.1926 |
| 1980-1984 | 1.0816 | 1.029 | 1.137 | 1.1973 | 1.082 | 1.325 |
| 1985-1989 | 1.0761 | 1.0295 | 1.125 | 1.2126 | 1.1286 | 1.303 |
| 1990-1994 | 1.0453 | 1.0036 | 1.0887 | 1.1309 | 1.08 | 1.1842 |
| 1995-1999 | 1 | 1 | 1 | 1 | 1 | 1 |
| 2000-2004 | 0.9742 | 0.9361 | 1.0139 | 0.9997 | 0.9555 | 1.046 |
| 2005-2009 | 0.9156 | 0.8773 | 0.9556 | 0.9242 | 0.8611 | 0.9918 |
| 2010-2014 | 0.867 | 0.8274 | 0.9084 | 0.8358 | 0.7567 | 0.9232 |
| 2015-2018 | 0.7491 | 0.7102 | 0.7901 | 0.7621 | 0.6696 | 0.8673 |
| Cohort |  |  |  |  |  |  |
| 1890 | 1.5227 | 1.271 | 1.8241 | 0.7018 | 0.6086 | 0.8092 |
| 1895 | 1.6283 | 1.4546 | 1.8228 | 0.92 | 0.8419 | 1.0052 |
| 1900 | 1.6915 | 1.552 | 1.8435 | 1.0847 | 1.0106 | 1.1643 |
| 1905 | 1.7443 | 1.6245 | 1.8729 | 1.2354 | 1.1614 | 1.3142 |
| 1910 | 1.6986 | 1.595 | 1.8088 | 1.3626 | 1.2871 | 1.4426 |
| 1915 | 1.6719 | 1.5792 | 1.7701 | 1.4689 | 1.3922 | 1.5497 |
| 1920 | 1.6804 | 1.5938 | 1.7717 | 1.5699 | 1.4915 | 1.6524 |
| 1925 | 1.5502 | 1.4731 | 1.6313 | 1.5548 | 1.4792 | 1.6343 |
| 1930 | 1.4162 | 1.346 | 1.49 | 1.4727 | 1.4015 | 1.5475 |
| 1935 | 1.3302 | 1.2635 | 1.4004 | 1.4224 | 1.3523 | 1.4961 |
| 1940 | 1.219 | 1.1582 | 1.2831 | 1.2862 | 1.2219 | 1.3538 |
| 1945 | 1.1493 | 1.0933 | 1.2083 | 1.2249 | 1.164 | 1.289 |
| 1950 | 1.0559 | 1.0046 | 1.1099 | 1.1518 | 1.0937 | 1.2129 |
| 1955 | 1 | 1 | 1 | 1 | 1 | 1 |
| 1960 | 0.9236 | 0.8732 | 0.977 | 0.9702 | 0.9107 | 1.0336 |
| 1965 | 0.9052 | 0.8487 | 0.9656 | 0.9258 | 0.8558 | 1.0016 |
| 1970 | 0.8351 | 0.7715 | 0.904 | 0.7653 | 0.685 | 0.8549 |
| 1975 | 0.7654 | 0.6907 | 0.8481 | 0.6825 | 0.5794 | 0.804 |
| 1980 | 0.7813 | 0.6917 | 0.8826 | 0.787 | 0.6344 | 0.9763 |
| 1985 | 0.7761 | 0.6698 | 0.8993 | 0.5734 | 0.4172 | 0.7882 |
| 1990 | 0.8743 | 0.7383 | 1.0353 | 0.6072 | 0.4064 | 0.9073 |
| 1995 | 0.8126 | 0.6493 | 1.0168 | 0.6235 | 0.3511 | 1.1074 |
| 2000 | 0.8158 | 0.5987 | 1.1116 | 0.5404 | 0.1995 | 1.4635 |
| 2005 | 1.1866 | 0.7714 | 1.8252 | 0.492 | 0.0745 | 3.2509 |
| 2010 | 0.8662 | 0.3108 | 2.414 | 0.6747 | 0.0061 | 74.1357 |
| 2015 | 1.8404 | 0.2158 | 15.6932 | 0.7867 | 0.001 | 601.2717 |

Abbreviations: IBM=incidence-based mortality, RR=rate ratio.
